# Fish oil and krill oil supplementations differentially regulate lipid catabolic and synthetic pathways in mice

**DOI:** 10.1186/1743-7075-11-20

**Published:** 2014-04-27

**Authors:** Veronika Tillander, Bodil Bjørndal, Lena Burri, Pavol Bohov, Jon Skorve, Rolf K Berge, Stefan EH Alexson

**Affiliations:** 1 Division of Clinical Chemistry, Department of Laboratory Medicine, Karolinska Institutet, Karolinska University Hospital, Stockholm, S-14186, Sweden; 2 Department of Clinical Science, University of Bergen, Bergen, N-5020, Norway; 3 Department of Heart Disease, Haukeland University Hospital, Bergen, N-5021, Norway; 4 Current address: Aker BioMarine Antarctica, Fjordalléen 16, Oslo, NO-0115, Norway

**Keywords:** Omega-3 fatty acids, Plasma lipids, High-fat diet, Gene regulation, Krill oil

## Abstract

**Background:**

Marine derived oils are rich in long-chain polyunsaturated omega-3 fatty acids, in particular eicosapentaenoic acid (EPA) and docosahexaenoic acid (DHA), which have long been associated with health promoting effects such as reduced plasma lipid levels and anti-inflammatory effects. Krill oil (KO) is a novel marine oil on the market and is also rich in EPA and DHA, but the fatty acids are incorporated mainly into phospholipids (PLs) rather than triacylglycerols (TAG). This study compares the effects of fish oil (FO) and KO on gene regulation that influences plasma and liver lipids in a high fat diet mouse model.

**Methods:**

Male C57BL/6J mice were fed either a high-fat diet (HF) containing 24% (wt/wt) fat (21.3% lard and 2.3% soy oil), or the HF diet supplemented with FO (15.7% lard, 2.3% soy oil and 5.8% FO) or KO (15.6% lard, 2.3% soy oil and 5.7% KO) for 6 weeks. Total levels of cholesterol, TAG, PLs, and fatty acid composition were measured in plasma and liver. Gene regulation was investigated using quantitative PCR in liver and intestinal epithelium.

**Results:**

Plasma cholesterol (esterified and unesterified), TAG and PLs were significantly decreased with FO. Analysis of the plasma lipoprotein particles indicated that the lipid lowering effect by FO is at least in part due to decreased very low density lipoprotein (VLDL) content in plasma with subsequent liver lipid accumulation. KO lowered plasma non-esterified fatty acids (NEFA) with a minor effect on fatty acid accumulation in the liver. In spite of a lower omega-3 fatty acid content in the KO supplemented diet, plasma and liver PLs omega-3 levels were similar in the two groups, indicating a higher bioavailability of omega-3 fatty acids from KO. KO more efficiently decreased arachidonic acid and its elongation/desaturation products in plasma and liver. FO mainly increased the expression of several genes involved in fatty acid metabolism, while KO specifically decreased the expression of genes involved in the early steps of isoprenoid/cholesterol and lipid synthesis.

**Conclusions:**

The data show that both FO and KO promote lowering of plasma lipids and regulate lipid homeostasis, but with different efficiency and partially via different mechanisms.

## Background

Omega-3 polyunsaturated fatty acids (PUFAs) such as eiocosapentaenoic acid (EPA) and docosapentaenoic acid (DHA) are well known bioactive dietary compounds that are found particularly in marine-derived food sources such as e.g. fatty fish, seaweed, shellfish, microalgae and krill. Since 1970, regular consumption of fish (preferably fatty fish) has been stated to have several positive effects on cardiovascular health [1-5]. The American Heart Association dietary guidelines for healthy individuals proposes consumption of at least two servings of fish per week which should yield an intake of approximately 400-500 mg EPA and DHA, and they recommend an even higher intake of omega-3 fatty acids to patients with documented coronary heart disease [6,7]. However, in large parts of the world the consumption of fish is considered to be inadequate and fish oil (FO) from anchovy, sprat, herring and salmon as a source of DHA and EPA has become widely used as a dietary supplement. The reported health benefits of FO have however led to an increased demand that may endanger natural resources of fish, and krill oil (KO) has recently emerged on the market as an alternative source of omega-3 PUFAs. Most FO on the market today have their omega-3 PUFAs incorporated into (triacylglycerols) TAG or in ethyl esters. However in KO are the majority of these omega-3 PUFAs esterified in phospholipids (PLs) [8-11]. KO has been stated to be a safe source of EPA and DHA that like other marine-based oils is able to efficiently raise the plasma levels of EPA and DHA [12-16]. However, the structural differences in the PUFA-rich lipid molecules may affect the distribution in cellular lipid fractions and tissue uptake and thereby promote different regulatory effects on lipid homeostasis [17]. KO also contains astaxanthin, which due to its anti-oxidative effect, might enhance the stability of the omega-3 PUFAs in the oil and thereby preserve them from lipid oxidation [10].

Intake of EPA and DHA has been shown to improve cardiovascular health by regulating lipid and glucose metabolism by acting as ligands for several nuclear transcription factors (e.g. peroxisome proliferator-activated receptors (PPARs) -α, -β/δ, and -γ and sterol regulatory element-binding protein 1 (SREBP-1)) [18-20]. EPA and DHA also have anti-inflammatory effects due to their conversion to less inflammatory signaling molecules at the expense of production of pro-inflammatory molecules from arachidonic acid (for reviews see [21,22]).

The liver is a central metabolic organ that regulates both circulating blood lipids and glucose levels by catabolism as well as synthesis of lipids and carbohydrates. Marine-derived omega-3 PUFAs have previously been shown to modulate the gene transcription profile in liver to enhance lipid degradation and decrease VLDL secretion (for review, see [23]). Recently, also KO was shown to modulate the transcriptional profile in mouse and rat liver and to affect plasma and liver lipids in mice [24-28].

Gene expression is regulated in the intestine in response to different metabolic conditions in order to cope with changes in nutrient load and content, to signal satiety and other stimuli to the rest of the body and to keep the intestinal defense barrier against pathogens intact. In addition, the intestine contributes to the plasma lipoprotein profile by absorbing lipids for chylomicron synthesis and being responsible for a significant part of the HDL production in the body [29,30]. To the best of our knowledge, so far no study has addressed the effects of KO on regulation of gene expression in the small intestine, although KO was recently shown to attenuate inflammation and oxidative stress in colon in an experimental rat model of ulcerative colitis [31].

The aim of this study was to compare the effects of two of the major sources of omega-3 supplements on the market today, FO and KO, when supplemented to a Western-like high-fat diet.

Equal amount of FO and KO (6% by weight) were added to a high fat diet, and the effects on plasma and liver lipids and gene regulation in liver and intestine were measured. In spite of the markedly lower omega-3 PUFA content in KO, both dietary supplementations raised the content of omega-3 PUFAs in plasma as well as in liver phospholipids to a similar extent. However, FO more efficiently lowered plasma lipids and this decrease was associated with accumulation of lipids in liver. In contrast, KO was less efficient in lowering plasma lipids with less, if any, sign of TAG accumulation in the liver. These different effects by FO and KO can at least in part be ascribed to differential regulation of gene expression in liver and intestine and different effects on VLDL secretion.

## Methods

### Animals and diets

Nine to ten week old male C57BL/6J mice were fed either a high-fat diet (HF) containing 24% (wt/wt) fat (21.3% lard and 2.3% soy oil, n = 9), or the HF diet supplemented with FO (EPAX 6000 TG®, a generous gift of Epax A/S, Ålesund, Norway) (15.7% lard, 2.3% soy oil and 5.8% FO, n = 6) or the HF diet supplemented with KO (Superba™, a generous gift of Aker BioMarine, Oslo, Norway) (15.7% lard, 2.3% soy oil and 5.7% KO, n = 6) and water *ad libitum* for 6 weeks. Diets were packaged in airtight bags and freeze stored until use to prevent lipid oxidation. Mice were housed in groups of three per cage at a constant temperature of 22 ± 2°C and a light/dark cycle of 12/12 h. Body weights of the animals were measured approximately every seventh day and food intake was measured three times in the beginning of the 6-week study to optimize the food supply. Animals were fasted overnight, anesthetized with 2% isoflurane (Schering-Plough, Kent, UK) and blood was collected by heart puncture. The blood was centrifuged and EDTA-plasma was frozen until further analysis. Livers were collected and the intestines were removed, rinsed with ice-cold phosphate buffered saline, cut into four segments of equal length that were further cut open and the epithelial cell layers were scraped off to separate the epithelial cells from the smooth muscle. All tissue samples were immediately frozen in liquid nitrogen and stored at -80°C until further analysis. The animal experiments were carried out with ethical permission obtained from the Norwegian State Board for Biological Experiments and followed the Norwegian Research Councils ethical guidelines.

### RNA isolation and cDNA synthesis

Total RNA from the intestinal epithelium and liver tissues was purified using the MagMax total RNA isolation system (Applied Biosystems, Carlsbad, CA, USA) after tissue homogenization. The quantity of the RNA was measured spectrophotometrically using a NanoDrop 1000 (NanoDrop Products, Wilmington, DE, USA) and the quality of the RNA was analyzed using the Experion Automated Electrophoresis System (Bio-Rad Laboratories, Hercules, CA, USA). The quality limit for further use of RNA was set to a R/Q value of ≥7 (out of 10). cDNA was synthesized with 500 ng RNA per reaction, using High Capacity RNA-to cDNA Mastermix (Applied Biosystems).

### Real-time PCR

Two types of TaqMan® Low Density Arrays in 96-well formats (format 96b, Applied Biosystems) were custom made to investigate the expression of genes related to peroxisomal and mitochondrial metabolic pathways, respectively (see Additional file 1 for gene lists). The plates were run at the Bioinformatics and Expression Analysis core facility (BEA) at Karolinska Institutet and the run data were analyzed by RQ Manager (Applied Biosystems). Gene expression was calculated using the 2**^**^**-ΔΔCt**^ method according to Livak et al. [32], using *18S* as reference gene and one individual sample in the high fat group as a calibrator (n = 4 per group). Some additional genes of interest (not being on the TLDA-plates) were analyzed in individual samples from liver and intestine (HF n = 6, FO n = 5, KO n = 6) using TaqMan or SYBR Green gene expression assays (for primers and additional TaqMan expression assays, see Additional file 1). SYBR Green primers were used at concentrations ranging from 100 to 200 nM and run with the Power SYBR Green Master Mix (Applied Biosystems). Again, gene expression data were calculated using the 2**^**^**-ΔΔCt**^ method (due to known efficiency of the primers used in the SYBR Green assay), however, the average Ct value of three different reference genes (*18S*, *Hprt* and *Ppia*) was used as control values and one individual sample in the high fat group as a calibrator.

### Lipid analysis

Liver lipids were extracted according to Bligh and Dyer [33], solvents were evaporated under nitrogen and the samples were re-dissolved in isopropanol before analysis. Lipids from liver extracts or plasma were then measured enzymatically on a Hitachi 917 system (Roche Diagnostics, Mannheim, Germany) using kits for analyzing total TAG (GPO-PAP kit, Roche Diagnostics), cholesterol (CHOD-PAP kit, Roche Diagnostics), total PLs (bioMérieux SA, Marcy l'Etoile, France) and NEFA (FS kit, DiaSyS, Holzheim, Germany). Aliquots of extracted liver lipids were separated by thin layer chromatography using silica gel plates (Merck, Darmstadt, Germany) and hexane:diethylether (1:1) as the liquid phase. The absolute levels of fatty acids of the diets, plasma and the TAG and PL fractions from livers were analyzed using gas chromatography as described previously by Grimstad et al. [31]. Lipoproteins were analyzed by size exclusion chromatography of plasma samples from individual mice (five mice in each group) according to Parini et al. [34].

### Hepatic enzyme activities

The liver tissue samples were homogenized and post-nuclear fractions were prepared as previously described [35]. Carnitine palmitoyltransferase 1 (CPT-I) activity was measured in the absence and presence of malonyl-CoA (15 μM) essentially as described by Bremer [36]. Peroxisomal acyl-CoA oxidase (ACOX) activity was determined by the coupled assay described by Small et al. [37] and fatty acid synthase (FAS) activity was measured as described by Skorve et al. [38].

### Statistics

Since normal distribution could not be assumed for the number of animal used in this study, the Kruskal-Wallis test was used for analysis of differences among the groups. If significance was obtained (*p* < 0.05) with the Kruskal-Wallis test, Dunn’s multiple comparison test was performed on all combinations, i.e. FO vs. KO, FO vs. high fat and KO vs. high fat. Significance indicated in tables and figures is shown for the Kruskal-Wallis test (as *p*-values in tables or as “K-W” in figures), and if significance was obtained in the Kruskal-Wallis test, also significance obtained by the post hoc test between the individual groups is indicated. A trend of difference between groups was set to 0.1 > *p* > 0.05 for the Kruskal-Wallis test. All values are presented as median and range. The statistics were calculated using GraphPad Prism 5.0d.

## Results

### Diet composition

The three different diets were isocaloric high-fat diets with 6% of the lard being exchanged for FO or KO. Although both FO and KO are rich in omega-3 PUFAs, there were differences in the fatty acid amount and composition between the two diets. The FO supplemented diet contained approximately double the amount of omega-3 PUFAs (about 3.7%, wt/wt) compared to the KO supplemented diet (about 1.8%, wt/wt). Instead the KO diet contained slightly more saturated (C14:0 and C16:0) and monounsaturated fatty acids than the FO diet (see Table 1 and Additional file 2).

**Table 1:** Diet composition

**Diet**	**High fat**	**FO**	**KO**
**Energy%**			
Protein	20.7	20.6	20.7
Fat	46.0	46.2	45.9
Carbohydrate	33.3	33.2	33.3
**Fat source** (% in diet)	Lard 21.3%	Lard 15.7%	Lard 15.6%
	Soy oil 2.3%	Soy oil 2.3%	Soy oil 2.3%
		Fish oil 5.8%	Krill oil 5.7%
**Fatty acids** (% of total fatty acids in diet)			
Total SFA	42.9	34.1	39.7
Total MUFA	38.7	32.1	35.4
Total ω-6 PUFA	16.4	14.5	14.6
Total ω-3 PUFA	1.9	19.1	10.1
EPA	0.03	8.97	5.23
DHA	0.05	6.40	2.28

FO; fish oil, KO; Krill oil, SFA; saturated fatty acids, MUFA; monounsaturated fatty acids, PUFA; polyunsaturated fatty acids.

### Body weight gain and liver weight

**Figure 1:**
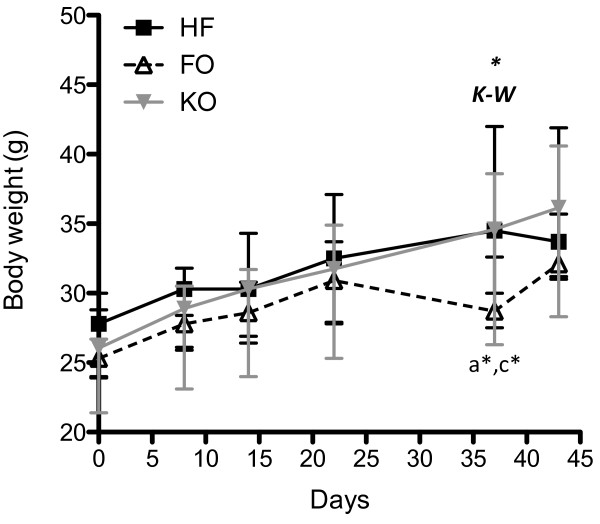
**Body weight gain.** The mice were weighed weekly and the figure shows body weights as median ± range of the animals in the three groups from day 0 to the endpoint at 6 weeks of feeding the different diets. Filled squares; HF (n = 9), open triangles; FO (n = 5), filled triangles; KO (n = 6). K-W* = significance by Kruskal-Wallis, a = HF vs. FO, c = FO vs. KO, * = p < 0.05 in Dunn post hoc test.

Body weights of the mice were not significantly different between the three groups at any of the time points except for a drop in the FO group at day 37 for unknown reasons (see Figure 1). Also the final weights of the animals were not significantly different (Table 2). One mouse in the FO group died early in the study, unlikely due to the treatment, reducing the number to five in this group. Food intake was only measured three times at the beginning of the study to optimize the food supply in order to minimize any potential oxidation of the PUFAs in the diet. These measurements did not indicate any difference in food intake in the two marine oil diet groups in comparison to the control group. No significant differences in liver weight or liver weight/body weight ratio between the groups were detected, although there was a trend towards a difference between the groups (p = 0.074, Kruskal-Wallis test) to higher liver weight/body weight in the FO group (Table 2).

**Table 2:** Final body and liver weights

	**High fat**	**FO**	**KO**	**K–W**
	**Median (range)**	**Median (range)**	**Median (range)**	***p***
Final body weight (g)	33.7 (31.0–41.9)	32.1 (31.2–35.7)	36.2 (28.3–40.6)	0.097
Liver weight (g)	1.65 (1.43–1.84)	1.74 (1.52–1.76)	1.66 (1.24–1.99)	0.851
Liver weight/body weight (%)	4.5% (4.3–5.7)	5.1% (4.7–5.5)	4.5% (3.9–5.0)	0.074

Values are shown as median with the range in brackets. High fat (n = 9), FO (n = 5) and KO (n = 6). K-W, *p* indicates the *p*-value with the Kruskal-Wallace test. FO; fish oil, KO; Krill oil.

### Plasma lipids and fatty acid composition

Total plasma cholesterol was significantly decreased by FO supplementation compared to the HF group (Table 3). This was due to significant decreases in both esterified and free cholesterol. Plasma TAG and PLs were significantly decreased in the FO group whereas non-esterified fatty acids (NEFA) were significantly decreased only in the KO group compared to HF. Notably, the post hoc test did not detect any significant differences between the two marine oil groups in any of these measurements.

**Table 3:** Plasma lipids

**Plasma lipids**	**High fat**	**FO**	**KO**	**K–W**
	**mmol/L**	**mmol/L**	**mmol/L**	***p***
Total cholesterol	3.08 (2.82–3.28)	2.05 (2.00–2.46)^a**^	2.59 (2.07–3.42)	0.007
Cholesterol esters	2.11 (1.88–2.32)	1.50 (1.36–1.80)^a*^	1.86 (1.27–2.44)	0.004
Free cholesterol	0.96 (0.92–0.99)	0.65 (0.50–0.66)^a**^	0.82 (0.68–0.98)	0.035
Triacylglycerol	1.12 (0.78–1.48)	0.49 (0.26–0.68)^a**^	0.77 (0.46–1.00)	0.008
Phospholipids	3.56 (2.87–3.95)	2.42 (2.17–2.52)^a**^	2.73 (2.27–3.26)	0.007
Nonesterified fatty acids	0.19 (0.14–0.29)	0.11 (0.02–0.14)	0.05 (0.00–0.18)^b*^	0.016

Data is shown as median and range. High fat (n = 5), FO (n = 5) and KO (n = 6). *a* = HF vs. FO, *b* = HF vs. KO, *= *p* < 0.05, **= *p* <0.01 by Dunn’s test. K-W*, p* indicates the *p*-value with the Kruskal-Wallace test. FO; fish oil, KO; Krill oil.

We further analyzed the distribution of cholesterol and TAG in the lipoprotein particles by size-exclusion chromatography to investigate whether marine oil supplementation changed the distribution of these lipids in the various lipoprotein fractions (Table 4). FO significantly lowered HDL and VLDL cholesterol while KO had no significant effect compared to HF. FO also strongly reduced VLDL cholesterol compared to the KO group. TAG was only significantly decreased in the VLDL fraction of the FO supplemented group. When analyzed by size-exclusion chromatography, the VLDL particles in FO fed mice eluted with the same retention time as in the HF group, suggesting that no major reduction in particle size had occurred (data not shown).

**Table 4:** Cholesterol and TAG content of the lipoprotein fractions in plasma

**Cholesterol**	**High fat**	**FO**	**KO**	**K-W**
	**mmol/L**	**mmol/L**	**mmol/L**	***p***
VLDL	0.11 (0.06–0.12)	0.04 (0.04–0.06)^a*, c*^	0.10 (0.07–0.12)	0.009
LDL	0.17 (0.14–0.61)	0.32 (0.27–0.37)	0.34 (0.17–0.51)	0.468
HDL	3.10 (2.42–3.36)	1.92 (1.71–2.21)^a*^	2.46 (1.85–3.15)	0.018
**Triacylglycerol**				
VLDL	0.79 (0.42–0.92)	0.20 (0.11–0.30)^a*^	0.37 (0.32–0.50)	0.004
LDL	0.27 (0.23–0.39)	0.23 (0.09–0.32)	0.20 (0.08–0.25)	0.063
HDL	0.08 (0.04–0.19)	0.06 (0.03–0.13)	0.08 (0.07–0.22)	0.133

Data is shown as median and range. High fat (n = 5), FO (n = 5) and KO (n = 5). *a* = HF vs. FO, *c* = FO vs. KO, *= *p* <0.05 by Dunn’s test. K-W*, p* indicates the *p*-value with the Kruskal-Wallace test. FO; fish oil, KO; Krill oil, VLDL; very low density lipoprotein, LDL; low density lipoprotein, HDL; high density lipoprotein.

The fatty acid composition was analyzed only in total plasma lipids due to limited amounts of sample. Quantitative fatty acid analysis showed a decrease in total fatty acids in both treatment groups with the difference being statistically significant in the FO group (Table 5). The decrease was due to decreased levels of total saturated (SFA) and monounsaturated (MUFA) fatty acids and omega-6 PUFAs but the amounts of omega-3 PUFAs were significantly increased in both groups. The decreased levels of SFA, MUFA and omega-6 fatty acids were due to decreased amounts of most fatty acid species. Although these effects were less pronounced in the KO group, KO significantly decreased arachidonic acid (C_20:4n-6_) compared to HF. Instead, FO strongly decreased the amount of C_18:2n-6_. The amount of omega-3 PUFAs increased in plasma to the same extent in both active treatment groups despite the markedly lower content of omega-3 PUFAs in KO. The relative abundance of the omega-3 PUFAs (EPA > DHA> > DPA > ALA) mirrored the composition in the respective marine oil supplemented diets rather closely, indicating that the omega-3 PUFA composition is not changed during intestinal absorption. However, the composition in plasma from the HF group was different with DHA being the dominating omega-3 PUFA (DHA> > EPA ≈ DPA ≈ ALA). Similar but less obvious trends were seen when comparing the relative fatty acid composition (in wt %), see Additional file 3.

**Table 5:** Total fatty acid composition of plasma lipids

**Fatty acids**	**High fat**	**FO**	**KO**	**K-W**
	**μg FA/ml plasma**	**μg FA/ml plasma**	**μg FA/ml plasma**	***p***
SFA	1255 (889–1928)	715 (570–731)^a**^	735 (611–925)	0.001
C10:0	0.9 (0.4–3.2)	1.0 (0.7–1.4)	1.8 (1.2–2.1)	0.126
C12:0	1.5 (0.6–2.9)	0.4 (0.3–0.6)^a**^	0.7 (0.5–1.0)	0.001
C14:0	15.3 (7.4–24.3)	5.8 (4.1–6.7)^a**^	11.4 (9.2–20.5)	0.003
C16:0	781 (524–1170)	422 (359–436)^a**^	482 (379–596)	0.002
C18:0	402 (311–637)	242 (181–271)^a**^	228 (187–274)^b**^	0.001
C20:0	11.0 (7.6–19.5)	4.6 (3.7–5.4)^a***^	6.4 (5.1–8.1)	0.003
C22:0	16.2 (10.6–26.6)	9.7 (7.7–10.3)	13.1 (10.7–17.3)	0.199
C24:0	5.0 (2.8–8.4)	3.9 (3.0–4.2)^a**^	3.8 (3.8–4.8)	0.001
MUFA	679 (493–1115)	277 (217–292)^a***^	351 (331–527)	0.001
C16:1n–9	11.7 (7.5–17.4)	4.4 (4.0–4.9)^a**^	5.7 (4.7–8.2)	0.001
C16:1n–7	45.2 (23.9–51.8)	18.9 (14.6–24.5)^a**, c*^	35.0 (24.7–53.0)	0.007
C18:1n–9	543 (397–919)	218 (170–232)^a***^	254 (241–390)	0.000
C18:1n–7	42.5 (33.0–70.5)	20.1 (14.3–22.7)^a**^	36.9 (30.6–45.1)	0.004
C20:1n–9	12.9 (8.7–24.8)	3.7 (2.9–4.8)^a***^	5.9 (4.6–7.6)	0.001
C20:1n–7	2.6 (1.7–4.2)	1.1 (0.8–1.4)^a**^	2.0 (1.8–2.5)	0.002
C22:1n–9	2.0 (1.3–4.1)	0.5 (0.4–0.6)^a*, c**^	3.0 (2.3–3.5)	0.004
C22:1n–7	1.4 (0.9–1.7)	0.6 (0.5–0.7)^c***^	2.1 (1.8–2.6)	0.000
C24:1n–9	9.6 (6.5–14.3)	7.2 (6.3–7.8)^a*^	7.5 (6.0–10.2)	0.040
ω-6 PUFA	1490 (1080–2176)	468 (419–527)^a***^	744 (513–815)	0.001
C18:2n–6	969 (677–1461)	283 (214–293)^a***^	562 (351–615)	0.000
C18:3n–6	14.1 (8.2–22.8)	2.6 (2.3–3.0)^a***^	4.8 (3.0–5.2)	0.001
C20:3n–6	48.4 (30.2–69.2)	12.8 (12.0–15.5)^a**^	15.0 (10.8–21.0)^b*^	0.001
C20:4n–6	455 (338–588)	189 (167–219)	136 (123–166)^b***^	0.000
C22:4n–6	5.8 (4.2–10.3)	1.1 (0.8–1.1)^a**^	1.2 (1.0–1.4)^b*^	0.001
C22:5n–6	7.7 (4.3–12.0)	3.1 (2.5–3.8)	1.3 (1.1–1.6)^b***^	0.000
ω**–**3 PUFA	272 (187–415)	773 (639–898)^a**^	696 (588–877)^b**^	0.001
C18:3n–3	18.3 (10.4–29.3)	3.8 (2.3–6.4)^a**^	11.3 (5.6–16.9)	0.002
C20:5n–3	21.4 (13.1–28.3)	420 (351–512)^a**^	400 (316–486)^b*^	0.001
C22:6n–3	212 (147–328)	261 (248–331)^a*^	268 (231–334)^b*^	0.013
C22:5n–3	14.1 (9.6–19.9)	31.2 (24.1–35.0)^a**^	26.0 (19.1–33.4)^b*^	0.002
ω**-**3/ω**-**6 PUFA	0.2 (0.2–0.2)	1.6 (1.5–1.7)^a***^	1.1 (0.8–1.4)	0.000
Total FA	3712 (2679–5648)	2282 (1849–2427)^a**^	2406 (2175–3079)	0.002

Quantitative fatty acid composition of selected fatty acids in plasma. SFA; saturated fatty acids, MUFA; monounsaturated fatty acids, PUFA; polyunsaturated fatty acids. Data are shown as median and range. High fat (n = 9), FO (n = 5), KO (=6). a = HF vs. FO, *p* <0.05, b = HF vs. KO, *p* <0.05, c = FO vs. KO, *= *p* <0.05, **= *p* <0.01 and ***= *p* <0.001. K-W, *p* indicates the *p*-value with the Kruskal-Wallace test. FO; fish oil, KO; Krill oil.

### Liver lipids and fatty acid composition

The hepatic levels of total cholesterol, PLs and TAG, as well as the fatty acid composition of the TAG and PL fractions were analyzed. Total cholesterol was significantly increased (≈25%) in both oil-supplemented groups compared to HF (Table 6). Furthermore, total PLs were significantly increased in the FO group, and also in the KO group when analyzed as total fatty acid content (see Tables 6 and 7). Total liver TAG content differed in the study population (Kruskal Wallis p = 0.045), although the post hoc test failed to identify significant differences between the three groups, probably due to one individual with extremely high TAG levels in the HF group. The extracted liver lipids were further separated by thin layer chromatography and quantitative fatty acid analysis of the TAG and PL fractions was performed, which showed that FO significantly elevated total fatty acid content in the TAG fraction compared to the HF group (Table 8). This increase was due to increases in some SFA species (specially palmitic acid (C_16:0_) and a >20-fold increase in omega-3 PUFAs. KO did not significantly affect total fatty acid amount, SFA, MUFA or omega-6 fatty acids but increased the omega-3 fatty acid content ≈ 8-fold. Notably, FO provoked a much stronger increase (≈3-fold) in omega-3 PUFA content in liver TAG compared to the effect of KO. In spite of the lower incorporation of omega-3 PUFAs in TAG, KO was more efficient in decreasing the amount of C_20_-C_22_ omega-6 fatty acids in this lipid fraction. Taken together these changes resulted in significantly increased omega-3/omega-6 ratios in both active treatment groups. For liver TAG fatty acid composition as wt %, see Additional file 4.

**Table 6:** Liver lipids

**Liver lipids**	**High fat**	**FO**	**KO**	**K-W**
**μmol/g**				***p***
Total Cholesterol	5.6 (4.8–6.7)	6.9 (6.0–10.4)a^*^	7.0 (5.9–9.7)b^*^	0.005
Triacylglycerol	12.2 (8.0–50.8)	42.2 (22.7–55.4)	17.0 (6.7–32.7)	0.045
Phospholipids	18.5 (16.3–20.8)	21.7 (21.5–23.5)a^**^	20.7 (19.2–22.6)	0.002

Data is shown as median and range. High fat n = 9, FO n = 5, KO n = 6. *a* = HF vs. FO, *b* = HF vs. KO, *= *p* <0.05, **= *p* <0.01 for Dunn’s test. K-W, *p* indicates the *p*-value with the Kruskal-Wallace test. FO; fish oil, KO; Krill oil.

**Table 7:** Fatty acid composition of the PL fraction in liver

**Fatty acids**	**High fat**	**FO**	**KO**	**K-W**
	**μg FA/g liver**	**μg FA/g liver**	**μg FA/g liver**	***p***
SFA	7174 (6094–8626)	9118 (8693–9471)^a**^	8918 (7882–9276)^b*^	0.001
C10:0	1.7 (0.9–3.8)	2.4 (1.3–2.7)	1.6 (0.6–3.0)	0.878
C12:0	1.8 (0.6–5.6)	4.7 (3.5–6.1)^a*^	2.5 (1.9–3.9)	0.022
C14:0	15.2 (10.1–17.4)	21.5 (19.5–23.3)^a**^	19.6 (17.4–27.4)^b**^	0.001
C16:0	3732 (3082–4211)	4990 (4560–5257)^a*^	5078 (4433–5170)^b**^	0.001
C18:0	2886 (2635–3975)	3816 (3682–4001)^a**^	3462 (3003–3650)	0.007
C20:0	72.3 (56.8–78.2)	72.4 (52.3–80.6)	85.8 (79.2–102)^b**^	0.005
C22:0	151 (130–169)	141 (98.4–142)^c***^	188 (155–225)	0.001
C24:0	52.8 (34.4–75.1)	69.5 (61.4–77.5)	66.2 (55.7–74.3)	0.179
MUFA	1732 (1473–2232)	2008 (1722–2546)	2168 (1996–2915)^b*^	0.012
C16:1n–9	25.6 (20.3–33.5)	28.6 (22.9–36.9)	31.0 (27.2–40.2)	0.063
C16:1n–7	94.7 (67.3–131)	141 (118–165)^a*^	169 (103–249)^b**^	0.003
C18:1n–9	1257 (1095–1638)	1506 (1231–1998)	1614 (1472–2162)^b*^	0.015
C18:1n–7	220 (174–295)	225 (211–260)	249 (217–276)	0.198
C20:1n–9	31.9 (27.0–50.6)	32.4 (32.2–37.6)	32.0 (30.8–45.2)	0.600
C20:1n–7	7.2 (5.6–10.5)	8.2 (6.7–9.3)	8.2 (7.2–13.3)	0.410
C22:1n–9	7.0 (5.5–8.5)	4.9 (4.8–6.6)^a*^	6.5 (5.4–7.0)	0.020
C22:1n–7	5.1 (3.8–6.5)	4.7 (3.4–4.9)^c**^	6.5 (5.5–9.2)	0.006
C24:1n–9	51.3 (40.6–77.3)	61.9 (57.1–63.9)	50.5 (44.4–78.0)	0.172
ω–6 PUFA	6206 (5254–7494)	3874 (3697–4144)^a***^	4768 (4056–5520)	0.001
C18:2n–6	2881 (2305–3231)	1935 (1677–2052)^a*, c**^	2865 (2449–3607)	0.004
C18:3n–6	45.4 (24.0–56.9)	16.6 (13.7–19.6)^a***^	24.6 (18.8–30.8)	0.001
C20:3n–6	286 (210–344)	163 (153–210)^a**^	174 (155–248)^b*^	0.001
C20:4n–6	2984 (2470–3728)	1808 (1576–1818)^a*^	1629 (1324–1723)^b***^	0.001
C22:4n–6	40.0 (31.8–60.4)	11.4 (9.3–12.0)^a**^	12.1 (10.1;13.7)^b*^	0.001
C22:5n–6	35.8 (27.2–67.8)	29.7 (27.1–35.2)	10.4 (9.6–11.6)^b***^	0.001
ω-3 PUFA	1931 (1824–2155)	6189 (5560–6985)^a***^	5660 (4417–6124)^b*^	0.001
C18:3n–3	18.4 (14.0–22.8)	19.2 (14.9–25.8)^c^	33.0 (27.6–39.8)^b**^	0.002
C20:5n–3	77.9 (52.0–83.8)	2057 (1621–2186)^a***^	1435 (1211–2261)^b*^	0.001
C22:6n–3	1774 (1666–1970)	3615 (3308–4480)^a**^	3612 (2614–3964)^b**^	0.001
C22:5n–3	74.7 (59.3–95.7)	321 (296–371)^a***^	276 (203–323)^b*^	0.001
ω–3/ω–6 PUFA	0.3 (0.3–0.4)	1.7 (1.3–1.7)^a***^	1.1 (0.9–1.3)^b*^	0.001
Total FA	17281 (15088–20534)	21171 (20619–22608)^a*^	21626 (18584–22927)^b**^	0.001

Quantitative fatty acid analysis of the most abundant fatty acids in the PL fraction from liver. SFA; saturated fatty acids, MUFA; monounsaturated fatty acids, PUFA; polyunsaturated fatty acids. Data are shown as median and range. High fat (n = 9), FO (n = 5), KO (n = 6). a = HF vs. FO, b = HF vs. KO, c = FO vs KO. *= *p* <0.05, **= *p* <0.01 and ***= *p* <0.001. K-W, *p* indicates the p-value with the Kruskal-Wallace test. FO; fish oil, KO; Krill oil.

**Table 8:** Fatty acid composition of the TAG fraction in liver

**Fatty acids**	**High fat**	**FO**	**KO**	**K-W**
	**μg FA/g liver**	**μg FA/g liver**	**μg FA/g liver**	***p***
SFA	2567 (1678–12724)	9625 (5443–13484)	3741 (1279–7260)	0.036
C10:0	1.9 (1.3–7.3)	8.0 (4.9–11.0)^a*^	3.1 (1.4–8.1)	0.019
C12:0	7.9 (2.0–59.8)	37.2 (28.2–43.0)^a*^	14.0 (7.2–26.3)	0.016
C14:0	41.3 (27.3–240)	161.1 (122–270)	82.6 (21.9–167)	0.063
C16:0	2067 (1367–11132)	8642 (4733–12151)^a*^	3196 (1041–6389)	0.029
C18:0	294 (182–823)	610 (360–815)	251 (136–372)^b*^	0.026
C20:0	89.3 (32.1–166)	115.0 (60.7–132)	58.7 (31.3–97.0)	0.158
C22:0	17.8 (5.9–30.6)	18.5 (11.8–21.1)	13.0 (7.2–21.6)	0.567
C24:0	3.9 (1.5–5.6)	6.9 (4.4–8.0)^c**^	3.0 (1.7–3.7)	0.006
MUFA	3659 (2367–19647)	12583 (5295–18215)	4388 (1458–11092)	0.095
C16:1n–9	69.8 (42.6–487)	264.0 (116–442)	94.3 (24.5–303)	0.145
C16:1n–7	113 (77–1190)	771 (475–1202)	360 (48.4–1058)	0.075
C18:1n–9	3088 (2016–16228)	10246 (4347–15384)	3595 (1252–8971)	0.095
C18:1n–7	171 (108–1160)	540 (221–694)	200 (72.3–459)	0.148
C20:1n–9	135 (64.9–362)	181 (75.9–319)	68.9 (31.2–158)	0.080
C20:1n–7	26.7 (12.8–98.0)	49.3 (20.2–72.4)	20.3 (8.3–44.4)	0.116
C22:1n–9	26.9 (12.2–48.6)	24.1 (12.6–40.6)	17.2 (7.2–29.6)	0.160
C22:1n–7	5.7 (2.3–12.5)	6.7 (3.7–8.9)	5.3 (2.1–9.2)	0.621
C24:1n–9	2.8 (1.3–3.2)	3.6 (2.5–5.3)^c*^	2.0 (1.1–2.2)	0.020
ω–6 PUFA	1751 (1290–7085)	2611 (1684–3193)	1851 (822–2969)	0.507
C18:2n–6	1418 (1029–5911)	2262 (1442–2781)	1677 (744–2724)	0.516
C18:3n–6	35.7 (28.5–105)	29.2 (16.2–40.0)	24.2 (10.5–36.7)	0.053
C20:3n–6	77.2 (42.0–222)	61.0 (32.9–83.2)	30.0 (15.0–51.9)^b**^	0.006
C20:4n–6	121 (99.2–542)	133 (111–165)^c*^	60.9 (28.4–88.5)^b**^	0.002
C22:4n–6	37.7 (27.4–137)	39.7 (24.8–48.3)^c*^	13.6 (7.8–19.0)^b**^	0.002
C22:5n–6	23.8 (15.6–56.4)	47.1 (35.4–56.3)^c***^	11.8 (6.2–17.0)^b*^	0.026
ω–3 PUFA	278 (210–1393)	6355 (4656–7148)^a***^	2192 (831–3543)	0.000
C18:3n–3	55.9 (34.6–291)	190 (129–197)	146 (41.2–265)	0.135
C20:5n–3	11.9 (9.8–113)	1329 (1170–2156)^a***^	594 (143–1048)^b*^	0.000
C22:6n–3	158 (127–688)	3097 (2200–3535)^a***^	1004 (496–1542)	0.000
C22:5n–3	41.0 (25.9–217)	1275 (814–1486)^a***^	330 (117–463)	0.001
ω–3/ω–6 PUFA	0.2 (0.2–0.2)	2.6 (2.0–2.8)^a***^	1.2 (0.9–1.5)^b*^	0.000
Total FA	8274 (5559–40955)	30744 (17101–41288)^a*^	12380 (4398–24899)	0.029

Quantitative fatty acid analysis of the most abundant fatty acids in the TAG fraction from liver. SFA; saturated fatty acids, MUFA; monounsaturated fatty acids, PUFA; polyunsaturated fatty acids. Data are shown as median and range. High fat (n = 9), FO (n = 5), KO (=6). a = HF vs. FO, b = HF vs. KO, c = FO vs. KO. *= *p* <0.05, **= *p* <0.01 and ***= *p* <0.001. K-W, *p* indicates the *p*-value with the Kruskal-Wallace test. FO; fish oil, KO; Krill oil.

Quantitative fatty acid analysis confirmed the increased fatty acid content in liver PLs by FO and further revealed a significantly increased fatty acid content in the PL fraction also by KO. This was due to increases in SFA, MUFA and omega-3 PUFAs in spite of decreased omega-6 fatty acid species, especially C_18:2_ by FO and C_20:4_ by KO and FO compared to HF (Table 7). The increased amount of SFA (25-30%) was mainly due to increases in C_16:0_ and C_18:0_. Interestingly, the EPA, DHA and DPA contents were quite similar in the FO and KO groups in spite of the lower omega-3 content in the KO diet. For liver PL fatty acid composition given in wt %, see Additional file 5.

### Effect of marine oils on gene expression in the liver

**Figure 2:**
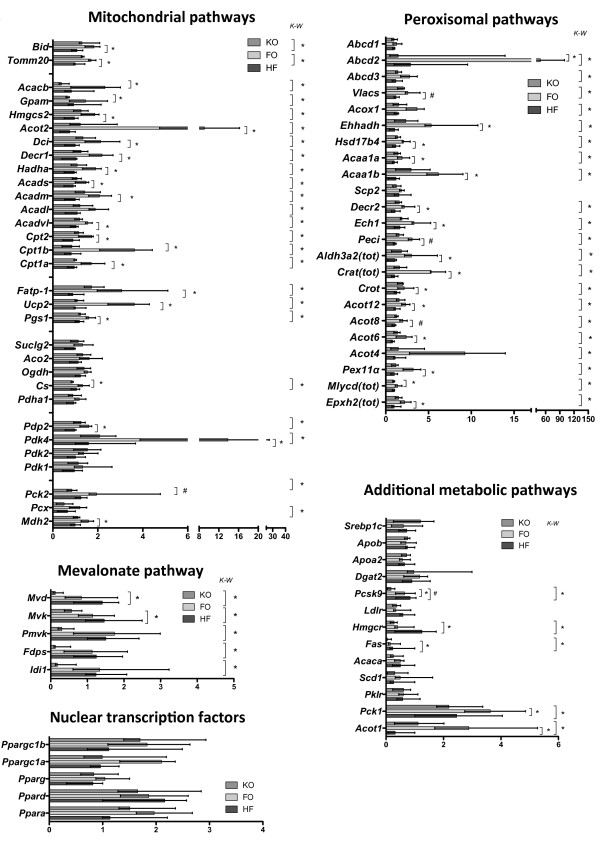
**Gene expression in liver.** The graphs showing the relative mRNA expression of genes coding for selected proteins in “Peroxisomal pathways”, “Mitochondrial pathways”, “Mevalonate pathway” and “Nuclear transcription factors” are generated from the TLDA array described in Materials and Methods. The bars show the median and range of the relative expression in the control group (HF, dark grey bars), the FO group (FO, light grey bars) and the KO group (KO, medium gray bars). Values are shown in relation to the expression of respective gene in one individual of the HF group (n = 4 per group). The graph showing “Additional metabolic pathways” was generated by real time PCR on selected genes as described in Material and Methods with the bars showing the median and the range of the relative mRNA expression in relation to one individual (set to 1) in the HF group. HF; dark grey bars (n = 6), FO; light grey bars (n = 5) KO; medium grey bars (n = 6). Significance by the Kruskal-Wallis test is shown to the right in each graph (K-W), and a star close to the bars show significance by the Dunn post hoc analysis (* = p < 0.05, # = p < 0.01 for the post hoc test).

Gene expression analysis was performed on genes coding for selected peroxisomal and mitochondrial proteins. The expression of several genes involved in fatty acid metabolism were upregulated in the FO group, e.g. genes involved in uptake (*Fatp-1*) and β-oxidation of fatty acids (*Vlacs, Acox1, Ehhadh, Hsd17b4, Acaa1b, Decr2, Ech1* and *Peci* in peroxisomes and *Cpt1a, Cpt1b, Hadha, Acadvl, Acadm, Acads, Decr1* and *Dci* in mitochondria) (Figure 2, see “Peroxisomal pathways” and “Mitochondrial pathways”) compared to HF. In line with the increased expression of *Acox1* mRNA, also peroxisomal acyl-CoA oxidase (ACOX) activity was significantly increased in the FO group. However, the increased mRNA expression of the two *Cpt1* genes did not translate into a significant increase of total CPT1 activity in the FO group (Table 9). In addition, a number of genes encoding enzymes that are involved in regulation of β-oxidation and transport of metabolites were upregulated by FO, e.g. acyl-CoA thioesterases (*Acot6*, *Acot8* and *Acot12* in peroxisomes and mitochondrial *Acot2*), as well as the short- and medium-chain carnitine acyltransferases *Crat* and *Crot*.

**Table 9:** Liver enzyme activities

**Diet**	**CPT1 activity**	**CPT1 activity**	**ACOX activity**	**FAS activity**
	nmol/mg/min - 15μM Malonyl-CoA	nmol/mg/min + 15μM Malonyl-CoA	nmol/mg/min	nmol/mg/min
**High fat**	2.35 (1.83–3.74)	1.24 (1.05–4.41)	16.8 (14.4–30.6)	0.31 (0.24–0.38)
**FO**	3.42 (2.22–3.73)	2.10 (1.61–2.20)	43.8 (33.3–54.7)^a**^	0.57 (0.36–0.63)^c***^
**KO**	2.50 (1.84–3.08)	1.49 (1.28–2.15)	22.4 (16.8–39.4)	0.20 (0.12–0.22)
*p* (K-W)	0.337	0.120	0.005	0.001

Acyl-CoA oxidase (ACOX), fatty acid synthase (FAS) and carnitine palmitoyltransferase 1 (CPT1) activities were measured as described in Materials and Methods. CPT1 activity was measured with and without the addition of 15 μM malonyl-CoA. Data shown as median and range. High fat (n = 6), FO (n = 5) and KO (n = 6). a = HF vs FO, c = FO vs KO. ^**^= *p* <0.01 and ^***^= *p* <0.001. K-W, *p* indicates the *p* -value with the Kruskal-Wallace test. FO; fish oil, KO; Krill oil.

Interestingly, KO decreased the expression of the mitochondrial-associated *Acacb* (acetyl-CoA carboxylase 2), which produces malonyl-CoA, compared to FO (Figure 2, “Mitochondrial pathways”). The decreased *Acacb* expression indicates a positive effect by KO on mitochondrial β-oxidation rate by reduced production of the CPT1-inhibitor malonyl-CoA.

Many of the genes that were upregulated by FO supplementation are well known targets for PPARα and involved in fatty acid degradation. Cytosolic acyl-CoA thioesterase 1 (*Acot1*) is one of the most strongly PPARα-regulated genes and a previously characterized target for PPARα [39]. *Acot1* expression was upregulated in the FO group and also *Hmgcs2* (mitochondrial rate limiting enzyme in ketone body formation) was upregulated in the FO group but not appreciably by KO (Figure 2, see “Additional metabolic pathways” and “Mitochondrial pathways”), suggesting a more potent PPARα-activation by FO.

The expression of the first enzymes of the cholesterol/isoprenoid synthesis pathway (*Mvd*, *Mvk*, *Pmvk*, *Fdps* and *Idi1*) did differ in the total study set, but the post hoc test only confirmed significant downregulation of *Mvd, Mvk* and *Hmgcr* by KO compared to HF (Figure 2, “Mevalonate pathway” and, “Additional metabolic pathways”), while no, or less pronounced decreases were found with FO supplementation. *Pcsk9*, a well-known regulator of degradation of the LDL receptor [40], was significantly downregulated by both KO and FO. No changes were found in the expression of lipoproteins *ApoB* or *ApoAII* or the TAG-synthesizing enzyme *Dgat2* by FO or KO. Fatty acid synthase (*Fas*) expression was significantly downregulated by KO, in line with the (non-significantly) lower activity of the enzyme (c.f. Table 9). The increased FAS-activity in the FO group was however not supported by the tendency to decreased *Fas* mRNA expression (see Table 9 and Figure 2, “Additional metabolic pathways”).

No major changes in the expression of genes coding for proteins in the citric acid cycle (Figure 2, “Mitochondrial pathways”), or oxidative phosphorylation (data not shown) were observed in either group compared to the HF group. However, *Pdk4*, a known PPARα responding gene, was strongly upregulated in the FO group but not in the KO group (Figure 2, “Mitochondrial pathways”). Soluble phosphoenolpyruvate carboxykinase 1 (*Pck1*) expression was increased in the FO group compared to the KO group, but not when compared to HF (Figure 2, “Additional metabolic pathways). The same pattern was seen for the expression of the mitochondrial gluconeogenic gene *Pck2,* as well as the citric acid synthase coding gene *Cs* (Figure 2, “Mitochondrial pathways”)*.* No major changes were seen in the expression of selected genes coding for proteins involved in apoptosis or in the metabolism of reactive oxygen species, except for increased expression of the pro-apoptotic *Bid* and uncoupling protein 2 (*Ucp2*) (see “Mitochondrial pathway”), a well-known PPARα target gene [41], and an increased expression of epoxide hydrolase (*Ephx2*, see “Peroxisomal pathways”) by FO compared to KO.

Expression was also investigated for members of the PPAR family of nuclear receptors, that are known to bind lipids as ligands and thereby respond to changes in lipid homeostasis, and the PPARγ coactivators 1a and 1b. However, no significant differences in the expression of these selected genes were found due to large variation of expression levels between individuals (Figure 2, “Nuclear transcription factors”). Although many PPARα regulated genes were upregulated in the FO group, *Pparα* itself was not significantly increased by FO or KO (p = 0.15 by Kruskal-Wallis test). *Ppargc1a* showed a trend (p = 0.058 by Kruskal-Wallis test) to be different between the groups. Expression of *Srebp1c* (sterol regulatory element binding protein-1c), which is a regulator of e.g. *Fas* and *Acaca*, was also investigated, however, contradictory to expectation no downregulation of the gene by KO was found (Figure 2, “Additional metabolic pathways”).

### Effect of marine oils on gene expression in small intestine

**Figure 3:**
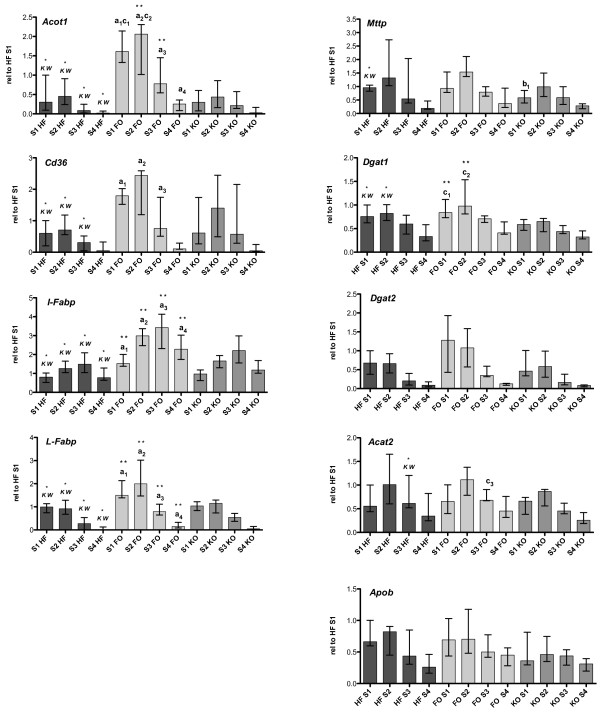
**Gene expression in small intestine.** mRNA expression of genes coding for proteins in fatty acid uptake and lipid synthesis in the intestinal epithelium of the four segments relative to the expression in segment 1 (S1) of one individual in the HF group. Graphs show the median expression value and range of each group. HF; dark grey bars (n = 6), FO; light grey bars (n = 5) KO; medium grey bars (n = 6). a_(segment)_ = HF vs. FO, p < 0.05; b_(segment)_ = HF vs. KO, p < 0.05; c_(segment)_ = FO vs. KO, **p < 0.01 for Dunn post hoc analysis after significant differences with the Kruskal-Wallis test, marked as K-W over respective segment in the HF group.

mRNA expression was investigated in the intestinal epithelium and the expression data are presented relative to the expression in the first segment of the small intestine to visualize both changes in expression and the expression pattern throughout the intestine. Gene expression of all fatty acid handling proteins investigated, *Cd36*, *I-Fabp,* and *L-Fabp* were significantly upregulated in all four segments of the small intestine, except for *Cd36* in segment 4 in FO supplemented mice (Figure 3). This observation is in line with the significant increase in *Acot1* that indicates that PPARα activation occurs also in the intestine of FO supplemented mice. However, none of these genes were significantly increased in the KO group. KO did not change the expression of any of the studied genes compared to HF except for a significant decrease in the expression of *Mttp* in the first segment of the intestine. However there were some significant differences seen between the FO and KO groups in that the proximal intestinal expression of *Dgat1* and the distal intestinal expression of *Acat2* were higher in the FO group*.* The expression of *Apob* and *Dgat2* was not significantly changed by either FO or KO. No changes in the expression of the cholesterol transporter *Npc1l1*, the efflux transporter *Abcg5* or the facilitated glucose transporter *Slc2a2* (*Glut2*) could be detected (data not shown).

## Discussion

Omega-3 PUFA supplementation and adequate intake of dietary omega-3 PUFAs is stated to have numerous beneficial health promoting effects including TAG lowering in plasma in humans, which have also been found in numerous animal studies [23]. The main outcome was the demonstration of a different metabolic regulation by KO and FO. FO significantly decreased several plasma lipid parameters (TAG, PLs and cholesterol) compared to HF, which was associated with lipid accumulation in liver. However, the post hoc test did not demonstrate any significant changes in total plasma TAG, PL or cholesterol in the KO supplemented group compared to HF, while plasma NEFA was significantly reduced. In addition, there were no significant differences between the two marine oil groups except for significantly lower VLDL cholesterol content in the FO group.

FO and KO differently regulated expression of genes involved in lipid degradation and synthesis. While FO provoked a strong PPARα activation like response in liver and intestine, these effects were weak, or absent, in the KO group, which instead mainly decreased the expression of genes involved in cholesterol and fatty acid synthesis.

The major factors contributing to these differences are likely the different content and structure of the omega-3 PUFAs in FO and KO. In this study we intentionally supplemented the high fat diet with equal amounts of oil (6% w/w) to compare the effects of commercially available omega-3 supplements. Although the omega-3 content is about double in FO, the concentration of omega-3 PUFAs in plasma was very similar between the two marine oil groups suggesting a higher bioavailability of KO, or that the content of omega-3 PUFAs in KO in our study is enough to obtain maximal plasma concentrations of omega-3 fatty acids. Incorporation of omega-3 PUFAs in PLs has been suggested to give these lipids a higher bioavailability [14,42,43], in fact, PLs enriched from KO was shown to be even more efficient in lowering plasma cholesterol than KO itself when given to rats fed a high cholesterol diet [44].

Unfortunately, in the present study was not enough plasma to analyze the fatty acid composition in TAG and PLs separately so it is not clear whether the similar levels of omega-3 PUFAs measured in plasma of the two groups are due to a specific enrichment in the PL fraction of the KO feed mice, which may be the case. However, the ratio of EPA/DHA (EPA > DHA) in total plasma was very similar in the FO and KO groups and rather closely resembled the ratio in the diet, indicating that these fatty acids are not modified during uptake in the intestine and transport in plasma. It is however evident that the omega-3 fatty acids are redistributed between the TAG and PL fractions following uptake and incorporation into liver lipids and that EPA is further metabolized to DHA, thereby markedly increasing the DHA/EPA ratio. DHA is mainly esterified into PLs in liver of the HF mice (constituting >90% of the omega-3 fatty acids in this fraction) and surprisingly reaching almost 50% of the DHA levels in the FO and KO groups. FO decreased total omega-6 fatty acids in total plasma lipids and liver PLs, while KO mainly lowered longer chain omega-6 fatty acid species in plasma and liver PLs. Both treatments thereby significantly increased the omega-3/omega-6 ratio in plasma compared to HF alone. Since KO specifically lowered the arachidonic acid content in the liver PL fraction and in plasma more efficiently than FO, KO may have a more potent anti-inflammatory effect. Such an effect by KO was seen in another FO and KO comparing supplementation study on rheumatoid arthritis with balanced amounts of DHA and EPA [45]. The decreased arachidonic acid levels are also likely to decrease the amounts of 2-arachidonoylglycerol (2-AG), a potent signaling lipid, as previously described [17]. Krill powder, which is also rich in omega-3 containing PLs, was shown to decrease another arachidonic acid derived endocannabinoid, anandamide (*N*-arachidonoylethanolamide, AEA), and its related metabolites palmitoylethanolamide (PEA) and oleoylethanolamide (OEA) in plasma of obese men [46]. KO has further been shown to be protective in a rat model of inflammatory bowel disease in which KO seemed to both act as an anti-inflammatory as well as anti-oxidative agent in the colon of these animals [31].

The plasma TAG lowering effect of EPA and DHA have been proposed to be at least partly mediated via PPARα, similar to fibrates, by stimulating the β-oxidation systems that would drain the liver of fatty acids and thereby decrease the production of VLDL particles. Our data, however, show a 3-4 fold TAG accumulation in livers from FO supplemented mice in spite of an apparent PPARα activation in both liver and intestine. Similar, but less pronounced, changes were seen also for fatty acids in plasma and liver PLs. The apparent ‘transfer’ of PLs from plasma to liver may reflect increased membrane synthesis, e.g. proliferation of peroxisomes (at least for the FO group) as indicated by increased expression of peroxisomal enzymes and mitochondria as indicated by slightly increased citrate synthase expression (see Figure 2). Another possibility is that liver PLs increase as a result of decreased HDL production that may at least in part explain the decrease in plasma HDL. Since fatty acid composition and amounts are similar in plasma and liver PLs in FO and KO supplemented mice, it is likely that the stronger PPARα activation seen in liver and intestine is mediated via omega-3 PUFAs of the TAG fraction of FO supplemented mice.

The increase in the fatty acid content of liver TAG found in the FO group indicates that the observed upregulation of genes coding for catabolic pathways (e.g. the upregulation of genes coding for fatty acid oxidation and the increased ACOX activity and possibly elevated activity of CPT1) is apparently of minor importance in determining liver lipid levels in these animals. It should be noted that PPARα activation by e.g. fenofibrate also increases liver expression of lipogenic genes a with concomitant increase in fatty acid synthesis and TAG accumulation [47]. Thus, our data are consistent with PPARα activation by FO, although weaker than fibrate treatment, and hence increased lipid synthesis and possibly decreased VLDL secretion may at least partially explain the observed TAG accumulation in liver. Also, a recent study demonstrated that DHA attenuates postprandial hyperlipidemia in a PPARα dependent manner by activation of fatty acid oxidation genes in the intestine leading to decreased TAG and apoB secretion. However, PPARα-independent pathways that reduce the assembly and secretion of VLDL particles also appear to be involved [23,48]. Some data indicate that FO decreases VLDL production mainly due to decreased plasma NEFA, which is suggested to be the main source of fatty acids for VLDL synthesis [23]. However only KO significantly decreased fasting plasma NEFA in our study but did not significantly change plasma TAG, suggesting additional mechanisms being involved. EPA is reported to inhibit DGAT activity in liver [49,50], and also negatively influence the assembly and secretion of VLDL via incorporation into choline and ethanolamine PLs, which due to the higher total content of EPA in FO could be an additional possible explanation for the different effects on plasma TAG by FO and KO in our study [51]. This may also explain why similar levels of omega-3 PUFAs were found in plasma, since the TAG pool entering the VLDL fraction might be affected by the high levels of EPA, and that EPA may be shunted into the cytosolic TAG-pool for storage rather than entering VLDL in the FO group*,* while the PL pool (which contained similar amounts of omega-3 PUFAs in both groups in liver) that enters the different lipoprotein fractions, e.g. HDL, may not be as affected [51]. We did not assess the severity of the lipid accumulation in liver in this study and therefore we can not predict the health effects associated with these changes and effects of long-term feeding of these omega-3 supplements.

KO feeding reduced the expression and the activity of fatty acid synthase (*Fas*), which is in line with previous findings that KO supplementation decreases the mRNA expression of this protein more efficiently than FO [25,27,28]*.* Expression of *Acacb* (acetyl-CoA carboxylase 2) was also decreased in the KO group compared to the FO group, which rather showed an increased expression of this gene. Acetyl-CoA carboxylase 2 is associated with mitochondria and generates malonyl-CoA, which is a key regulator of energy homeostasis [52], and deletion of *Acacb* in mice promotes fatty acid oxidation and increased energy expenditure [53,54]. Therefore, in conjunction with an apparent downregulation of *Fas* (and possibly *Acaca*) and thereby fatty acid synthesis in the KO group, this may provide a possible alternative mechanism by which KO maintains lower liver TAG levels compared to FO.

KO also reduced the mRNA expression of the first segments of the cholesterol/isoprenoid synthesis pathway, including the rate-limiting enzyme in cholesterol synthesis HMG-CoA reductase. However, the reduction in expression seen in our current study did not reflect in significant changes in plasma cholesterol (-16%), and liver cholesterol was even slightly increased as in the FO group. This was also seen in a low fat/omega-3 PUFA balanced fish and krill oil based study in which KO caused a similar downregulation in expression of genes involved in gluconeogenesis and cholesterol and fatty acid synthesis without changing plasma lipids [24]. Similar amounts of KO supplementation have previously been shown to decrease plasma and liver cholesterol in C56BL/6 mice on a HF diet, however in this study the mice were fed a different high fat diet containing buttermilk and 0.15% cholesterol ± KO for 8 weeks which may explain the different results [26]. More experiments are needed to elucidate the discrepancy between gene expression and plasma/liver cholesterol levels, e.g. measurement of total body cholesterol pool and bile acid and cholesterol synthesis and excretion.

In the present study we have also investigated the effects of FO and KO on gene expression of proteins involved in lipid metabolism in the small intestine. A similar trend in gene regulation as in the liver could be seen in the intestine of these animals. The expression of genes involved in fatty acid modulation and transport, such as *Acot1, Cd36, I-fabp* and *L-fabp* were all upregulated by the FO-containing diet, which is in line with previous findings of PPARα activation in the intestine during increased ligand availability [55]. KO on the other hand did not promote the same response to these PPARα driven genes. The increased expression of fatty acid transporters in the intestine of the FO fed group would potentially lead to an enhanced chylomicron production due to increased uptake of fatty acids, however FO has previously been shown to cause PPARα activation and thereby increased β-oxidation in murine intestine [56], which may balance an increased uptake of fatty acids. In a recent study, fenofibrate was shown to reduce blood TAG content in the postprandial state in part due to decreased dietary fat absorption and increased β-oxidation of fatty acids in spite of upregulation of chylomicron synthesizing genes and fatty acid transporters [57]. It is possible that the different effects of FO and KO may in part be due to differences in PPARα activation and thereby oxidative degradation of fatty acids in the small intestine, although the quantitative importance of fatty acid oxidation in the intestine is likely to be small. The only significant changes found in the KO group in genes coding for proteins in the chylomicron synthesis pathway was a downregulation of *Mttp* in the first intestinal segment by KO compared to HF diet, and a significant difference between FO and KO in the expression of *Acat2* (in segment 3) and *Dgat1* (in segments 2 and 3). Feces were not collected for absorption studies or lipid analysis in our study, therefore it is not clear whether omega-3 PUFAs per se, or omega-3 PUFAs in TAG versus PLs, affect dietary lipid absorption in the small intestine. Some data indicate that FO may reduce lipid uptake in the intestine, or at least delay efflux into the circulation by a transient accumulation of lipids in the enterocytes [58]. Whether KO affects lipid uptake in the intestine remains to be studied.

## Conclusions

Both FO and KO raised plasma levels of omega-3 PUFAs to the same extent in spite of a markedly lower omega-3 PUFA content in the KO diet. FO lowered plasma TAG, PL and cholesterol and KO lowered NEFA in comparison to the control group. The two omega-3 fatty acid supplementations also promoted different gene expression profiles in liver and intestine with FO causing an apparent PPARα response by increasing the expression of genes coding for proteins in the two β-oxidation systems and other lipid metabolizing genes. In contrast, KO supplementation rather acted as a negative regulator of endogenous cholesterol and fatty acid synthesis, at least at the mRNA level. The stronger plasma lipid lowering effect with FO can be partly explained by increased lipid accumulation, mainly as TAG, in liver in spite of increased PPARα activation that may not compensate for decreased VLDL secretion. The physiological/pathological implications of the liver lipid accumulation by FO are not clear and may depend on diet composition and dose of FO and should be evaluated.

## Abbreviations

ALA: Alpha-linolenic acid; ApoB: Apolipoprotein B; EPA: Eicosapentaenoic acid; DHA: Docosahexaenoic acid; FA: Fatty acid; FO: Fish oil; HDL: High density lipoprotein; KO: Krill oil; LDL: Low density lipoprotein; MUFA: Monounsaturated fatty acid; NEFA: Nonesterified fatty acids; PLs: Phospholipids; PPAR: Peroxisome proliferator-activated receptor; PUFA: Polyunsaturated fatty acid; TAG: Triacylglycerol; VLDL: Very low density lipoprotein.

## Competing interest

Lena Burri is currently an employee at Aker BioMarine Antarctica. There is no other conflict of interest reported.

## Authors’ contribution

VT carried out the gene expression analysis, participated in the extraction of liver and plasma lipids and in the plasma lipoprotein determination, analyzed and compiled results and drafted the manuscript. PB carried out the fatty acid composition analyses on diets and tissues and contributed to the manuscript. LB and BB compiled and interpreted results, drafted the manuscript and together with VT and SEHA participated in the animal experiments. RKB, JS, LB and SEHA designed and coordinated the study, as well as participated in the interpretation of data and in completing the manuscript. All authors read and approved the final manuscript.

## Supplementary Material

Additional file 1: **List of primers used in the experiment.** SYBR Green primer sequences and Taqman gene expression assays. Click here for file

Additional file 2: **Fatty acid composition of diets.** The most abundant fatty acids in the respective diets are shown as % of total fatty acids. Click here for file

Additional file 3: **Fatty acid composition of total plasma lipids.** The most abundant fatty acids in plasma are shown as % of total fatty acids. Click here for file

Additional file 4: **Fatty acid composition of liver TAG fraction.** The most abundant fatty acids in the liver TAG fraction are shown as % of total fatty acids. Click here for file

Additional file 5: **Fatty acid composition of liver PL fraction.** The most abundant fatty acids in the liver PL fraction are shown as % of total fatty acids. Click here for file
