# Supplementary material for: Fish oil and krill oil supplementations differentially regulate lipid catabolic and synthetic pathways in mice
Source: Nutr Metab (Lond). 2014 Apr 27;11:20. doi: 10.1186/1743-7075-11-20 (PMC4021563; doi:10.1186/1743-7075-11-20)
Supplement: Additional file 1: — List of primers used in the experiment. SYBR Green primer sequences and Taqman gene expression assays. [file 1743-7075-11-20-S1.pdf]

Additional file 1

Primer list for additional genes quantitated by real-time PCR

**SYBR Green primers**	5' to 3'	3' to 5'
Acaca	fw acctgtacaagcagtgtgggct	rev cacatggcctggcttggaggg
Acat2	fw tgtccctgtctttgccaaca	rev catgcaagatggagagcagc
Abcg5	fw cgcggacttctacaacaaga	rev gggggagaaggagagctg
Acot1	fw ctggcgcatgcaggatc	rev ggcacttttcttggatagctcc
Apob	fw tcagtgaaggtaccaaccgga	rev tgacgtggacttggtgctctc
Asbt	fw agctggtcaaccctggtaca	rev gggggagaaggagagctg
Cd36	fw ttgtacctatactgtggctaaatgaga	rev cttgtgttttgaacatttctgctt
Cyclophilin	fw gatgagaacttcatcctaaagcataca	rev tcagtcttggcagtgcagataaa
Fas	fw ggcatcattgggcactcctt	rev gctgcaagcacagcctctct
Hmg-CoA red	fw ccggcaacaacaagatctgtg	rev atgtacaggatggcgatgca
Hprt	fw ggtgaaaaggacctctcgaagtg	rev atagctaagggcatatccaacaac
I-fabp	fw acggaacggagctcactg	rev ttaccagaaacctctcggaca
Ldlr	fw gcatcagcttggacaaggtgt	rev gggaacagccaccattgttg
L-fabp	fw ccatgactggggaaaaagtc	rev gcctttgaaagttgtcaccat
Ncp1l1	fw tacacggcctggtcttcct	rev cagtaccagagcttggttaacatc
Psck9	fw cagggctggaatgcaaaatc	rev gccacagtgacctgctctga
Srebp1	fw ggagccatggattgcacatt	rev ggcccgggaagtcactgt

**Taqman expression assays**	
18S	4310893E-0211020 (VIC-TAMRA)
Apoa2	Mm00442687_m1
Dgat1	Mm00515643_m1
Dgat2	Mm00499530_m1
Mttp	Mm00435015_m1
Pck1	Mm00440636_m1
Pdk4	Mm00443325_m1
Pklr	Mm00443090_m1
Slc2a2	Mm00446224_m1

Taqman expression assays for the two custom made Taqman low density arrays (TLDA).

Abcd1-Mm00431749_m1<br>Abcd2-Mm00496455_m1<br>Abcd3-Mm00436150_m1<br>Acaa1a-Mm00728460_s1<br>Acaa1b-Mm00728805_s1<br>Acacb-Mm01204691_m1<br>Acadl-Mm00599660_m1<br>Acadm-Mm00431611_m1<br>Acads-Mm01203498_m1<br>Acadvl-Mm00444296_m1<br>(Acnat1)RP23-34B24.1-Mm01624101_s1<br>(Acnat2)C730036D15Rik-Mm01626730_s1<br>Aco2-Mm00475673_g1<br>Acot12-Mm00499641_m1<br>Acot2-Mm01622461_s1<br>Acot3-Mm00652967_m1<br>Acot4-Mm00506680_m1<br>Acot6-Mm01297748_m1<br>Acot8-Mm00504683_m1<br>Acox1-Mm00443579_m1<br>Acox2-Mm00446408_m1<br>Acox3-Mm00446122_m1<br>Acsl4-Mm00490331_m1<br>Aldh3a2-Mm00839320_m1<br>Amacr-Mm00507717_m1<br>Atp5e-Mm00445969_m1<br>Atp5g1;Gm10039-Mm01303239_gH<br>Baat-Mm00476075_m1<br>Bax-Mm00432051_m1<br>Bcl2l1-Mm00437783_m1<br>Bid-Mm00432073_m1<br>Cat-Mm00437992_m1<br>Cox10-Mm00617695_m1<br>Cox4i1-Mm00438289_g1	Cpt1a-Mm00550438_m1<br>Cpt1b;Chkb-cpt1b-Mm00487200_m1<br>Cpt2-Mm00487205_m1<br>Crat-Mm00483985_m1<br>Crot-Mm00470079_m1<br>Cs-Mm00466043_m1<br>Dci-Mm00494452_m1<br>Decr1-Mm00470689_m1<br>Decr2-Mm00496393_m1<br>Ech1-Mm00469322_m1<br>Ehhadh-Mm00470091_s1<br>Ephx2-Mm00514706_m1<br>Esrra-Mm00433143_m1<br>Fdps-Mm00836315_g1<br>Gpam-Mm00833328_m1<br>Gpx1-Mm00656767_g1<br>Gstk1-Mm00504022_m1<br>Hacl1-Mm00517379_m1<br>Hadha-Mm00805228_m1<br>Hmgcs2-Mm00550050_m1<br>Hsd17b4-Mm00500443_m1<br>Hspa1b-Mm03038954_s1<br>Hspd1-Mm00849835_g1<br>Hspe1-Mm00434083_m1<br>Idi1-Mm00836417_g1<br>Mcl1-Mm01257351_g1<br>Mdh2-Mm00725890_s1<br>Me2-Mm00521023_m1<br>Mvd-Mm00507014_m1<br>Mvk-Mm00445773_m1<br>Ndufs2-Mm00467603_g1<br>Nfe2l2-Mm00477784_m1<br>Nrf1-Mm00447996_m1<br>Nrip1-Mm01343437_m1<br>Nudt19-Mm00473613_m1<br>Nudt7-Mm00452560_m1	Ogdh-Mm00803121_m1<br>Pck2-Mm00551411_m1<br>Pcx-Mm01239167_m1<br>Pdha1-Mm00468675_m1<br>Pdk1-Mm00554306_m1<br>Pdk2-Mm00446681_m1<br>Pdk3-Mm00455220_m1<br>Pdk4-Mm00443325_m1<br>(Pdp2)4833426J09Rik-Mm01252669_s1<br>Peci-Mm00478725_m1<br>Pecr-Mm00502709_m1<br>Phyh-Mm00477734_m1<br>Pmvk-Mm00503429_m1<br>Ppara-Mm00440939_m1<br>Ppard-Mm01305434_m1<br>Pparg-Mm00440945_m1<br>Ppargc1a-Mm01208832_m1<br>Ppargc1b-Mm00504720_m1<br>Prdx3-Mm00545848_m1<br>Prdx5-Mm00465365_m1<br>Scp2-Mm01257982_m1<br>Sdha-Mm01352366_m1<br>Slc25a1-Mm01171197_g1<br>(Fatp-1)Slc27a1-Mm00449511_m1<br>(Vlacs)Slc27a2-Mm00449517_m1<br>Sod2-Mm01313000_m1<br>Suclg2-Mm01182166_g1<br>Txn2-Mm00444931_m1<br>Txnrd2-Mm00496774_m1<br>Ucp2-Mm00627599_m1<br>Ucp3-Mm01163394_m1<br>(Upc4)Slc25a27-Mm00511820_m1<br>Uqcrfs1-Mm00481849_m1<br>18S-Hs99999901_s1
