# Supplementary material for: Fish oil and krill oil supplementations differentially regulate lipid catabolic and synthetic pathways in mice
Source: Nutr Metab (Lond). 2014 Apr 27;11:20. doi: 10.1186/1743-7075-11-20 (PMC4021563; doi:10.1186/1743-7075-11-20)
Supplement: Additional file 2: — Fatty acid composition of diets. The most abundant fatty acids in the respective diets are shown as % of total fatty acids. [file 1743-7075-11-20-S2.pdf]

Additional file 2

Fatty acid composition of the diets. The most abundant fatty acids in respective diet and omega-3 PUFAs are shown in % of total fatty acids of diets.

Fatty acids (%)	High fat diet	Fish oil diet	Krill oil diet
C14:0	1.53	1.20	3.31
C16:0	24.8	19.4	23.0
C18:0	15.4	12.4	12.1
C16:1n-7	1.62	1.53	2.60
C18:1n-7	2.33	2.35	3.08
C18:1n-9	33.5	26.7	28.3
C18:2n-6	15.6	13.1	13.7
C18:3n-3	1.66	1.29	1.61
C20:5n-3	0.03	8.97	5.23
C22:6n-3	0.05	6.40	2.28
C22:5n-3	0.10	1.19	0.22
Other FA	3.31	5.52	4.55
