# Supplementary material for: Fish oil and krill oil supplementations differentially regulate lipid catabolic and synthetic pathways in mice
Source: Nutr Metab (Lond). 2014 Apr 27;11:20. doi: 10.1186/1743-7075-11-20 (PMC4021563; doi:10.1186/1743-7075-11-20)
Supplement: Additional file 3: — Fatty acid composition of total plasma lipids. The most abundant fatty acids in plasma are shown as % of total fatty acids. [file 1743-7075-11-20-S3.pdf]

Additional file 3

Fatty acid composition of total plasma lipids in %. Median values (range) are shown for SFA (saturated fatty acids), MUFA (monounsaturated fatty acids) and ω-6 and ω-3 PUFA (polyunsaturated fatty acids).

Fatty acids	High fat<br>g FA/100 g FA	Fish oil<br>g FA/100 g FA	Krill oil<br>g FA/100 g FA
**SFA**	**33.8 (32.5-34.5)**	**31.0 (29.9-31.3)**	**30.0 (28.1-30.6)**
C10:0	0.02 (0.01-0.10)	0.05 (0.04-0.06)	0.07 (0.05-0.08)
C12:0	0.04 (0.02-0.07)	0.02 (0.02-0.02)	0.03 (0.02-0.03)
C14:0	0.40 (0.27-0.49)	0.25 (0.22-0.29)	0.44 (0.42-0.67)
C16:0	21.0 (19.3-21.6)	19.1 (17.4-19.8)	18.9 (17.4-20.1)
C18:0	10.8 (10.3-11.6)	10.6 (9.76-11.5)	8.59 (8.31-9.48)
C20:0	0.30 (0.25-0.35)	0.22 (0.19-0.23)	0.24 (0.23-0.27)
C22:0	0.44 (0.37-0.47)	0.40 (0.36-0.56)	0.50 (0.44-0.56)
C24:0	0.14(0.09-0.20)	0.17(0.13-0.21)	0.16(0.13-0.17)
**MUFA**	**18.7 (17.5-22.8)**	**12.0 (11.4-13.6)**	**15.8 (13.8-17.1)**
C16:1n-9	0.31 (0.28-0.35)	0.21 (0.17-0.24)	0.25 (0.20-0.27)
C16:1n-7	1.07 (0.88-1.29)	0.84 (0.79-1.04)	1.61 (1.03-1.72)
C18:1n-9	15.2 (14.2-18.8)	9.25 (8.73-11.0)	11.5 (10.0-12.7)
C18:1n-7	1.22 (1.11-1.51)	0.85 (0.72-0.93)	1.46 (1.41-1.54)
C20:1n-9	0.39 (0.32-0.50)	0.16 (0.15-0.21)	0.21 (0.21-0.25)
C20:1n-7	0.07 (0.06-0.08)	0.05 (0.04-0.06)	0.08 (0.07-0.08)
C22:1n-9	0.06 (0.05-0.08)	0.02 (0.02-0.03)	0.11 (0.10-0.12)
C22:1n-7	0.03 (0.03-0.04)	0.03 (0.03-0.03)	0.08 (0.07-0.09)
C24:1n-9	0.25 (0.21-0.40)	0.32 (0.27-0.39)	0.26 (0.24-0.47)
**ω-6 PUFA**	**40.2 (37.2-41.3)**	**21.8 (21.3-22.7)**	**26.5 (23.6-31.1)**
C18:2n-6	25.9 (24.8-26.9)	12.0 (11.6-12.5)	20.2 (16.1-25.1)
C18:3n-6	0.35 (0.25-0.55)	0.12 (0.11-0.13)	0.17 (0.14-0.20)
C20:3n-6	1.17 (1.05-1.44)	0.61 (0.52-0.76)	0.51 (0.45-0.76)
C20:4n-6	12.4 (9.16-14.1)	8.48 (8.29-9.90)	5.32 (5.01-6.27)
C22:4n-6	0.17 (0.14-0.21)	0.05 (0.04-0.05)	0.05 (0.04-0.05)
C22:5n-6	0.17 (0.12-0.33)	0.13 (0.13-0.16)	0.05 (0.04-0.06)
**ω-3 PUFA**	**7.12 (6.12-8.22)**	**34.4 (33.9-37.0)**	**28.5 (24.4-32.0)**
C18:3n-3	0.50 (0.38-0.62)	0.18 (0.12-0.28)	0.39 (0.26-0.55)
C20:5n-3	0.51 (0.44-0.66)	19.4 (18.4-21.4)	15.5 (13.1-18.4)
C22:6n-3	5.73 (4.53-6.57)	13.2 (11.1-13.8)	10.9 (9.59-11.9)
C22:5n-3	0.37 (0.31-0.43)	1.37 (1.30-1.44)	1.02 (0.79-1.10)
**ω-3/ω-6 PUFA**	0.18 (0.16-0.20)	1.55 (1.52-1.72)	1.08 (0.79-1.39)
