# Supplementary material for: Fish oil and krill oil supplementations differentially regulate lipid catabolic and synthetic pathways in mice
Source: Nutr Metab (Lond). 2014 Apr 27;11:20. doi: 10.1186/1743-7075-11-20 (PMC4021563; doi:10.1186/1743-7075-11-20)
Supplement: Additional file 4: — Fatty acid composition of liver TAG fraction. The most abundant fatty acids in the liver TAG fraction are shown as % of total fatty acids. [file 1743-7075-11-20-S4.pdf]

Additional file 4

Fatty acid composition of liver TAG fraction in %. Median values (range) are shown for SFA (saturated fatty acids), MUFA (monounsaturated fatty acids) and ω-6 and ω-3 PUFA (polyunsaturated fatty acids).

Fatty acids	High fat<br>g FA/100 g FA	Fish oil<br>g FA/100 g FA	Krill oil<br>g FA/100 g FA
**SFA**	**31.18(28.27-32.44)**	**31.83(31.31-34.26)**	**29.12(28.09-32.03)**
C10:0	0.02 (0.02-0.03)	0.03 (0.02-0.03)	0.03 (0.02-0.04)
C12:0	0.09 (0.04-0.15)	0.11 (0.09-0.20)	0.11 (0.08-0.16)
C14:0	0.56 (0.40-0.69)	0.60 (0.51-0.79)	0.64 (0.50-0.84)
C16:0	26.1 (23.0-27.3)	28.1 (27.2-30.0)	25.0 (23.7-27.8)
C18:0	3.27 (2.01-3.85)	2.10 (1.69-2.38)	2.00 (1.50-3.08)
C20:0	0.79 (0.40-1.14)	0.36 (0.32-0.45)	0.57 (0.29-0.76)
C22:0	0.17 (0.05-0.25)	0.06 (0.04-0.08)	0.12 (0.05-0.23)
C24:0	0.03 (0.01-0.07)	0.02 (0.01-0.04)	0.02 (0.01-0.04)
**MUFA**	**44.2 (41.1-48.0)**	**36.78 (31.0-44.1)**	**35.4 (32.2-44.6)**
C16:1n-9	0.84 (0.71-1.19)	0.77 (0.68-1.07)	0.78 (0.56-1.22)
C16:1n-7	1.72 (1.19-3.12)	2.78 (2.20-3.51)	2.74 (1.10-4.25)
C18:1n-9	37.2 (35.1-39.6)	29.9 (25.4-37.3)	29.4 (27.0-36.7)
C18:1n-7	2.13 (1.83-2.83)	1.64 (1.29-1.76)	1.64 (1.52-1.85)
C20:1n-9	1.17 (0.81-1.64)	0.53 (0.44-0.77)	0.62 (0.51-0.71)
C20:1n-7	0.24 (0.20-0.32)	0.14 (0.12-0.18)	0.17 (0.14-0.19)
C22:1n-9	0.24 (0.11-0.38)	0.09 (0.07-0.10)	0.13 (0.10-0.21)
C22:1n-7	0.04 (0.03-0.08)	0.02 (0.02-0.03)	0.04 (0.03-0.07)
C24:1n-9	0.02 (0.01-0.04)	0.01 (0.01-0.02)	0.01 (0.01-0.03)
**ω-6 PUFA**	**21.2 (17.3-23.2)**	**8.15 (7.73-9.98)**	**14.7 (10.8-20.8)**
C18:2n-6	17.3 (14.4-18.6)	7.10(6.74-8.65)	13.30(9.83-19.22)
C18:3n-6	0.39 (0.26-0.64)	0.09 (0.07-0.13)	0.21 (0.14-0.30)
C20:3n-6	0.75 (0.54-0.93)	0.20 (0.19-0.24)	0.25 (0.20-0.34)
C20:4n-6	1.46 (1.15-2.01)	0.48 (0.32-0.65)	0.52 (0.36-0.64)
C22:4n-6	0.44 (0.27-0.56)	0.13 (0.12-0.15)	0.11 (0.08-0.18)
C22:5n-6	0.25 (0.13-0.36)	0.15 (0.14-0.21)	0.09 (0.07-0.14)
**ω-3 PUFA**	**3.48 (3.30-4.11)**	**20.9 (15.4-27.2)**	**17.8 (14.2-19.2)**
C18:3n-3	0.71 (0.62-0.78)	0.57 (0.46-0.75)	1.03 (0.86-1.63)
C20:5n-3	0.18 (0.14-0.28)	6.01 (2.87-7.77)	4.39 (3.26-5.17)
C22:6n-3	1.80 (1.66-2.54)	10.1 (7.93-13.5)	8.46 (6.19-11.3)
C22:5n-3	0.50 (0.47-0.60)	4.34 (3.39-4.88)	2.45 (1.86-3.17)
**ω-3/ω-6 PUFA**	0.17 (0.16-0.20)	2.62 (1.99-2.77)	1.17 (0.88-1.53)
