# Supplementary material for: Fish oil and krill oil supplementations differentially regulate lipid catabolic and synthetic pathways in mice
Source: Nutr Metab (Lond). 2014 Apr 27;11:20. doi: 10.1186/1743-7075-11-20 (PMC4021563; doi:10.1186/1743-7075-11-20)
Supplement: Additional file 5: — Fatty acid composition of liver PL fraction. The most abundant fatty acids in the liver PL fraction are shown as % of total fatty acids. [file 1743-7075-11-20-S5.pdf]

Additional file 5

Fatty acid composition of liver PL fraction in %. Median values (min and max values) are shown for SFA (saturated fatty acids), MUFA (monounsaturated fatty acids) and ω-6 and ω-3 PUFA (polyunsaturated fatty acids).

Fatty acids	High fat g FA/100 g FA	Fish oil g FA/100 g FA	Krill oil g FA/100 g FA
**SFA**	**41.4 (40.2-43.1)**	**42.4 (41.9-43.6)**	**41.1 (39.7-42.4)**
C10:0	0.01 (0.00-0.02)	0.01 (0.01-0.01)	0.01 (0.00-0.01)
C12:0	0.01 (0.00-0.03)	0.02 (0.02-0.03)	0.01 (0.01-0.02)
C14:0	0.09 (0.07-0.10)	0.10 (0.09-0.11)	0.10 (0.08-0.12)
C16:0	21.2 (20.3-22.5)	22.4 (21.7-24.2)	23.2 (22.0-24.1)
C18:0	17.8 (16.7-19.4)	17.9 (17.6-18.8)	15.9 (14.7-16.4)
C20:0	0.42 (0.35-0.47)	0.35 (0.25-0.38)	0.41 (0.35-0.47)
C22:0	0.85 (0.71-1.10)	0.63 (0.46-0.68)	0.88 (0.67-1.03)
C24:0	0.31 (0.21-0.41)	0.33 (0.30-0.34)	0.31 (0.29-0.34)
**MUFA**	**10.5 (9.32-11.2)**	**9.74 (8.18-12.0)**	**10.2 (9.34-12.7)**
C16:1n-9	0.15 (0.13-0.19)	0.13 (0.11-0.17)	0.14 (0.12-0.20)
C16:1n-7	0.49 (0.41-0.76)	0.65 (0.52-0.80)	0.86 (0.46-1.11)
C18:1n-9	7.76 (6.98-8.24)	7.16 (5.85-9.43)	7.47 (6.88-9.43)
C18:1n-7	1.27 (1.13-1.43)	1.03 (1.00-1.20)	1.15 (1.08-1.20)
C20:1n-9	0.20 (0.18-0.25)	0.16 (0.14-0.17)	0.15 (0.14-0.20)
C20:1n-7	0.04 (0.04-0.05)	0.04 (0.03-0.05)	0.04 (0.03-0.06)
C22:1n-9	0.04 (0.03-0.05)	0.02 (0.02-0.03)	0.03 (0.03-0.04)
C22:1n-7	0.03 (0.02-0.04)	0.02 (0.02-0.02)	0.03 (0.03-0.04)
C24:1n-9	0.33(0.23-0.38)	0.30(0.25-0.30)	0.23(0.21-0.34)
**ω-6 PUFA**	**36.5 (34.2-37.3)**	**18.2 (17.8-19.1)**	**22.2 (20.7-25.8)**
C18:2n-6	15.7 (14.7-16.9)	9.08 (8.13-9.32)	13.5 (12.0-16.9)
C18:3n-6	0.25 (0.15-0.37)	0.08 (0.07-0.09)	0.11 (0.08-0.14)
C20:3n-6	1.71 (1.39-2.13)	0.77 (0.70-0.97)	0.80 (0.69-1.16)
C20:4n-6	17.5 (16.1-19.2)	8.38 (7.45-8.61)	7.43 (7.12-7.74)
C22:4n-6	0.26 (0.21-0.30)	0.05 (0.04-0.06)	0.06 (0.05-0.06)
C22:5n-6	0.22 (0.17-0.33)	0.14 (0.13-0.16)	0.05 (0.05-0.06)
**ω-3 PUFA**	**11.8 (10.4-12.6)**	**30.0 (25.6-30.9)**	**25.5 (23.8-26.7)**
C18:3n-3	0.11 (0.09-0.12)	0.09 (0.07-0.11)	0.15 (0.13-0.19)
C20:5n-3	0.45 (0.34-0.51)	9.66 (7.47-10.6)	6.76 (5.66-9.86)
C22:6n-3	10.6 (9.5-11.7)	17.5 (15.6-19.8)	16.7 (14.1-18.6)
C22:5n-3	0.44 (0.37-0.52)	1.52 (1.40-1.64)	1.21 (1.09-1.48)
**ω-3/ω-6 PUFA**	0.33 (0.28-0.37)	1.67 (1.34-1.70)	1.14 (0.92-1.29)
